# Conditioned place preference successfully established in typically developing children

**DOI:** 10.3389/fnbeh.2015.00187

**Published:** 2015-07-20

**Authors:** Leah Ticker Hiller, Sandy Takata, Barbara L. Thompson

**Affiliations:** ^1^Mrs. T.H. Chan Division of Occupational Science and Occupational Therapy, University of Southern CaliforniaLos Angeles, CA, USA; ^2^Department of Cell and Neurobiology, University of Southern CaliforniaLos Angeles, CA, USA; ^3^Department of Pediatrics, Childrens Hospital of Los Angeles, Keck School of Medicine, University of Southern CaliforniaLos Angeles, CA, USA

**Keywords:** conditioned place preference, associative learning, child, affective, social behavior

## Abstract

Affective processing, known to influence attention, motivation, and emotional regulation is poorly understood in young children, especially for those with neurodevelopmental disorders characterized by language impairments. Here we faithfully adapt a well-established animal paradigm used for affective processing, conditioned place preference (CPP) for use in typically developing children between the ages of 30–55 months. Children displayed a CPP, with an average 2.4 fold increase in time spent in the preferred room. Importantly, associative learning as assessed with CPP was not correlated with scores on the Mullen Scales of Early Learning (MSEL), indicating that CPP can be used with children with a wide range of cognitive skills.

## Introduction

The development of cognitive functioning in children has been the subject of in-depth studies since the late 1800’s with descriptive milestones serving as the basis for measures of typical cognitive development. Atypical development of cognitive performance often is manifested as impairments and challenges in daily life for the infant and toddler, and thus evident to family, caregivers and health care professionals. Validated instruments for cognitive performance are routinely used, with diagnoses and subsequent interventions applied.

In contrast, affective processing, which can influence attention, motivation/reward, and emotional regulation, is a more difficult construct to test in young children. This is because determining affective state and adaptive capacities is often dependent on intact language skills. Key experiments have revealed important components of emotional development in children (as early as infancy), by relying on recognition tasks and facial expression of emotions (LaBarbera et al., 1976; Bowlby, 1977; Izard, 1978; Kagan, 1982; Kagan and Snidman, 1991). Less understood are the internal states determining affect, which directly impact, and possibly even drive specific behavioral output of children. Surveys, questionnaires, and expressive language can measure emotional affect and processing in verbal children, but the challenge is far greater early in development or when trying to determine these functions in a non-verbal child, which is prevalent in a variety of neurodevelopmental disorders (Luyster et al., 2008, 2011; Grossman and Tager-Flusberg, 2012; Kasari et al., 2013). Though challenging to assess, skill sets in domains influenced by affective processing are crucial for establishing capacity in developing executive function, an integrative set of abilities that greatly influence adaptive capacities, problem-solving, and academic and practical skill building (Davidson et al., 2006; Blair and Diamond, 2008; Diamond, 2011).

New strategies and tools that probe more complex internal responses, such as feelings, drives, and motivations independent from language, become necessary for populations of children with language delays and other language impairments, and even typically developing children. According to the Centers for Disease Control, neurodevelopmental disorders affect 15% of children in the United States (Boyle et al., 2011). Heterogeneity within each of the disorders, as well as medical and mental health conditions associated with neurodevelopmental disorders further complicates an understanding of mechanisms that underlie the behavioral phenotypes. Therefore, a need exists for unique experimental strategies to analyze emotional drives in children, independent from language development.

Here, we report on a unique translation of a research strategy from animals to children, utilizing conditioned place preference (CPP), a time-honored animal paradigm to measure differences in motivation, reward, and aversion. To our knowledge, this is the first attempt at establishing the use of CPP in children. In animals, CPP is routinely used to differentiate between rewarding, non-rewarding, and aversive stimuli, thereby probing internal affective state independent from language capacity (Garcia et al., 1957; Mucha et al., 1982; Spyraki et al., 1982; Bozarth, 1990; Panksepp and Lahvis, 2007). The current study addressed whether young children could learn a CPP. The paradigm utilizes straightforward Pavlovian conditioning methods to assess whether a preference has been conditioned. In this paradigm, a conditioned stimulus (CS) is repeatedly paired with an unconditioned stimulus (US), which elicits an unconditioned response (UR). With successful conditioning, the CS elicits a CR similar to the UR. The paradigm is sufficiently sensitive to reveal even modest differences in motivation, reward, and aversion. We reasoned that measured CRs could allow for a child’s specific actions to reflect affective state, rather than relying on expressive language, and thus would serve as a powerful tool for use in typically developing children or those with neurodevelopmental disorders.

While not done in children, there is a sound basis for such an approach. CPP has been used successfully in human adults (Childs and de Wit, 2009; Molet et al., 2013; Astur et al., 2014). US for these studies have included d-amphetamine, music, and food, although in the latter study, CPP could not be established without prior food deprivation in the subjects. Successful conditioned place aversion was also demonstrated in response to different music stimuli. In the present study, we adapted the CPP paradigm for use in children by using age-appropriate toys as the US, and a custom-designed child-friendly arena, a castle, as the CS. The experimental paradigm has a number of advantages for application to toddlers and young children in a single testing session, making it scalable for a variety of research populations. Furthermore, children perform associative learning frequently and very early in development (Coyle et al., 2000; Herbert et al., 2003; Heathcock et al., 2004; Minda et al., 2008; Preissler, 2008), and performance in the task is expected to be quite robust with conditioning parameters correctly established.

## Materials and Methods

### Participants

Twelve typically developing children, seven female and five male, between the ages of 2.5 and 5 years participated in this study. The Institutional Review Board of the University of Southern California, USA, approved the recruitment, caregiver consenting, and experimental protocols. Researchers monitored for adverse events, fatigue, and distress during the experiments. Caregivers were instructed they could stop, take a break, or discontinue the study at any point. The CPP task required that the child be physically able to traverse between the two sides of the arena and also separate easily from the caregiver. Participants were recruited using flyers advertising the general features of the study from multiple locations throughout the greater Los Angeles community including the University of Southern California, USA. Inclusion criteria were typically developing children with an age of 30–60 months. Exclusion criteria for subjects were the presence of severe sensory or motor impairments, absence of identified metabolic, genetic, or progressive neurological disorders, no family history of autism spectrum disorder (ASD) or intellectual disability in first-degree relatives, no psychological, emotional, or neurological diagnosis or any type of cognitive impairment or learning disorder (e.g., Down syndrome, dyslexia), and no known developmental delays as reported by the parents. Families were compensated for their participation with a monetary gift card.

### Assessments

#### Mullen Scales of Early Learning (MSEL; Mullen, 1995)

Each child was administered the Mullen Scales of Early Learning (MSEL). The MSEL is a play-based assessment designed to measure cognitive ability and motor development. It is a standardized assessment with five sub-scales: Gross Motor (only for children younger than 33 months), Visual Reception, Fine Motor, Expressive Language and Receptive Language. An early learning composite was derived from the *t*-scores of the four cognitive scales (per MSEL Manual instructions) for each child. The MSEL was performed in an assessment room equipped with one-way mirrors to allow parents to observe their child while the assessments were performed.

### Conditioned Place Preference (CPP)

#### Arena

**Figure 1:**
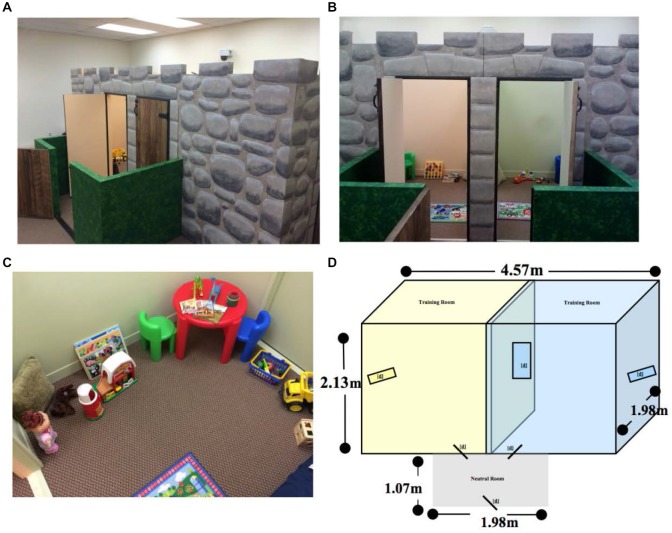
**Pictures of the conditioned place preference (CPP) arena. (A)** The newly constructed child-friendly, three room, custom-designed arena was designed as a castle to encourage children to explore the arena. **(B)** When standing in the neutral room, the child has to decide which of the two rooms they want to enter first. **(C)** Overhead view of one room in the castle arena. **(D)** Blueprint design of the physical dimensions of the custom-designed arena. [d] = door.

The CPP paradigm was performed in a custom-designed, three-room arena decorated as a castle (Figure 1). Within this arena, there were two unique training rooms connected to a smaller neutral room. Each training room was 2.13 m long by 2.28 m wide by 1.98 m tall. The connecting neutral room was 1.98 m long by 1.07 m wide by 76.2 cm tall. Each room was uniquely decorated by color and other visual cues (rugs, pillows, tables, and chairs), and therefore easily distinguishable by the child. Each room was outfitted with books, puzzles, dolls, and additional age-appropriate toys to engage children during the initial and final preference tests. The castle was contained within a room in the laboratory to minimize extraneous auditory and visual stimuli. Ceiling and wall mounted video cameras (Noldus Information Technology, Wageningen, Netherlands) allowed for continuous video monitoring in all parts of the arena during the training and testing sessions. These cameras were synchronized with Noldus Observer XT Software to allow for off-line behavioral coding and quantification.

#### Procedure

After completion of the MSEL, subjects were shown the outside of the arena and told they would play in the castle. Subjects freely explored the arena for approximately 2 min with the experimenter and parent/guardian for acclimation to the sounds and novelty of the arena. The child then exited the arena and the timed part of the experiment initiated.

#### Initial Preference Test

The doors to each room were opened, and the child was free to enter the arena. Once the child entered one of the training rooms from the neutral room, the doors connecting each training room to the neutral room were closed, while leaving the connecting door between the two training rooms open and the initial preference test began. Each of the conditioning rooms contained identical toys, books, and puzzles. The only distinctions between the two rooms during the initial preference test were the color and decorations within each room. The child was provided 6 min to explore both training rooms and an initial preference test score was calculated (see “Data Analysis” Section). After the initial preference test, the doors were opened, and the child exited the arena to play with an experimenter and different toys outside of the castle for approximately 2 min.

#### Training Trials

While the child played outside the castle, additional stimuli (toys and books) were placed in each room by the research team. For this study, the US were very engaging toys (musical toys, manipulatives accompanied by auditory sounds, etc.) or less engaging toys (puzzles and books). The CS were the uniquely decorated training rooms within the castle. The presentation of the US within each room was randomized and counterbalanced across subjects. The child was then given access to only one room of the arena with either the more or less engaging US, by closing the door separating the two training rooms. The child was then given access to the other room of the arena. Each training session was 3 min per room, with a total of four training sessions per room, alternating between rooms, in an A-B-A-B manner. The order of room presentation was also randomized and counterbalanced across subjects. After the last training session, the child again exited the castle and played with an experimenter and additional toys outside the castle for approximately 2 min.

#### Final Preference Test

While the child played outside the castle, the conditioning stimuli were removed from each room, and the baseline toys present during the initial preference test were placed back into the rooms for the final preference test. Additionally, the door separating the two training rooms was opened to allow access to both rooms during the preference test. The child was then allowed to enter the arena. Once the child entered one of the training rooms from the neutral room, the doors connecting each training room to the neutral room were closed, and the final preference test began. The child was allowed to freely explore both training rooms for an additional 6 min.

The duration of the visit for each participating child and their families was approximately 2 h, with a total of approximately 40 min for running the habituation and CPP paradigm. This allowed for each child to only make a single visit to the laboratory, thereby limiting the amount of time and effort required by the family to participate in the research study.

## Data Analysis

Multiple experimenters blind to the identity of the engaging toy-paired side of the arena scored videos for time spent on each side of the arena for each subject during the initial preference and final conditioning test trials. To determine initial preference, percent time spent on each side was calculated. To determine final preference, percent time on each side was calculated during the final preference test. An additional calculation was performed to determine CPP, while accounting for potential room bias. The CPP calculation is a percent change score: ((A2−A1)/A1)*100, where (A2) represents percent time spent on the most preferred side during the final preference test and (A1) represents the percent time spent on this same side during the initial preference test. The CPP score was also calculated as fold change score, as (A2/A1) for graphing purposes. Paired two-tailed, one sample *t*-tests, and correlation analyses were performed to determine statistical significances differences (*p* < 0.05) dependent upon the data comparisons (see “Results” Section). Inter-rater reliability was performed on 20% of randomly selected videotaped sessions. Kappa was greater than 0.89 with 95% CI.

## Results

### Demographics

A total of twelve typically developing children, seven female and five male ranging in age from 30–55 months, with a mean age of 42.8 months, participated in this study. MSEL sub-scale scores and early learning composite scores are reported in Table 1. There was a wide range of sub-scale scores, with the expressive language sub-scale showing the largest spread of scores.

**Table 1:** **Demographics and MSEL scores for the twelve (seven female and five male) participants**.

	Mean ± SD	Range
Chronological age (mos)	42.8 ± 10.8	30–55
MSEL Visual Reception age equivalent (mos)	43.6 ± 11.8	25–66
MSEL Fine Motor age equivalent (mos)	42.7 ± 12.2	23–59
MSEL Receptive Language age equivalent (mos)	42.2 ± 10.4	26–57
MSEL Expressive Language Age equivalent (mos)	45.3 ± 16.7	20–77
MSEL Early Learning Composite	101 ± 17.9	77–154

### Initial Preference Test

**Figure 2:**
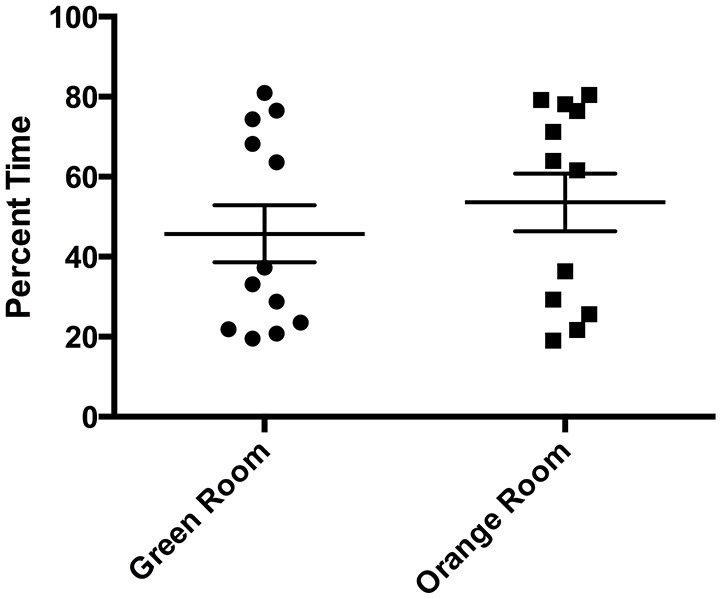
**Initial preference test room time**. There were no significant differences in percent time spent in each room during the initial preference test (*p* > 0.05). Individual data points are plotted with the Mean ± SEM.

Comparing percent time spent in each room during the initial preference test, we found no differences in mean time spent in each room (*t_11_* = 0.5479, *p* > 0.05) prior to conditioning (Figure 2). The lack of significant differences in time spent by subjects between rooms prior to conditioning confirmed that the paradigm design was effective in assuring that both rooms were equally salient, without eliciting a preference bias or in being aversive.

### Final Preference Test

**Figure 3:**
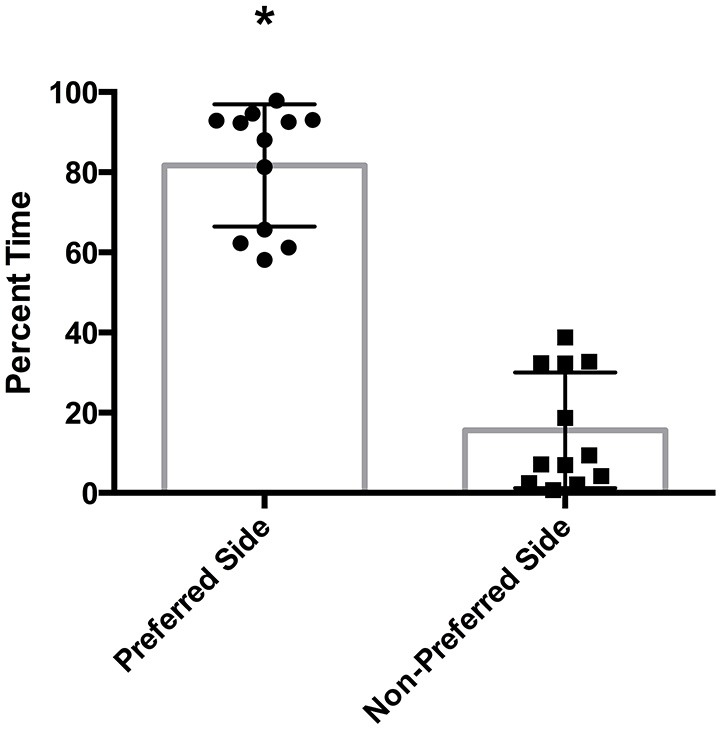
**Preferred room during final preference test**. Subjects displayed a significant preference for one room during the final preference test (*p < 0.001). Individual data points are plotted with the Mean ± SEM.

**Figure 4:**
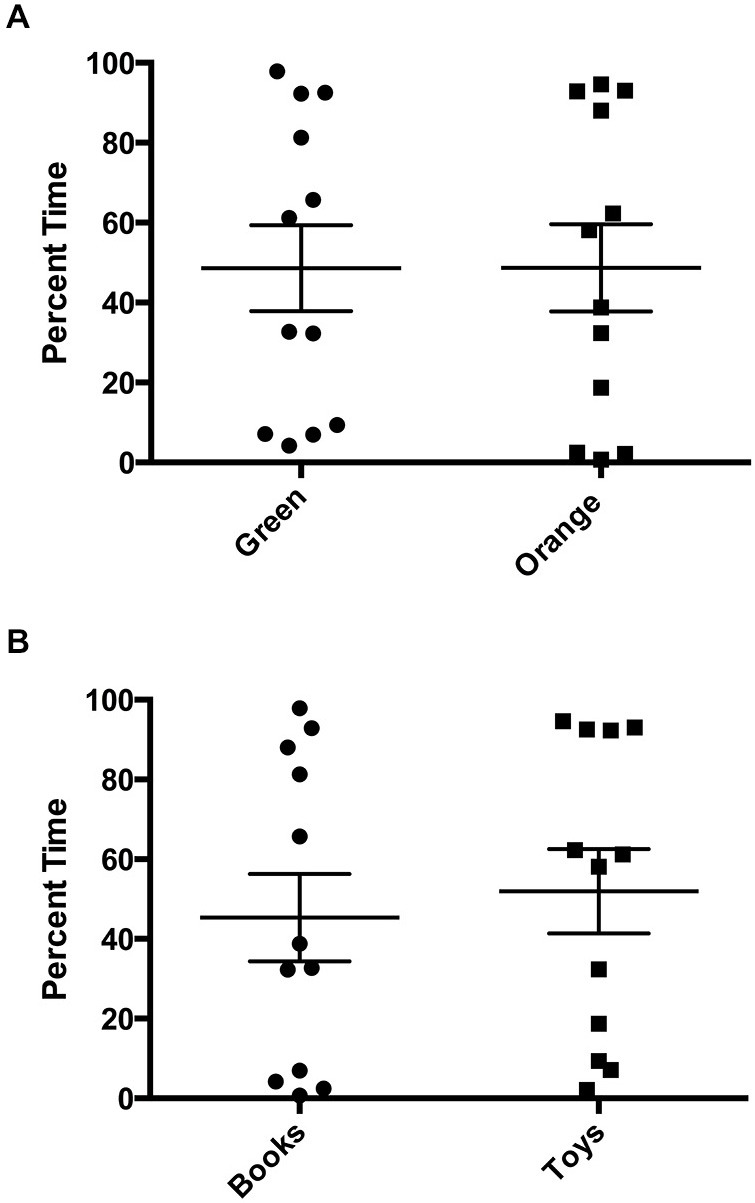
**Final preference test times. (A)** Independent from which unconditioned stimulus (US) was used for training, there were no significant differences in percent time spent in each room during the final preference test (*p* > 0.05). **(B)** Independent from which room was paired with the US, there were no significant differences in percent time spent with US (books vs. toys), (*p* > 0.05). Individual data points are plotted with the Mean ± SEM.

**Figure 5:**
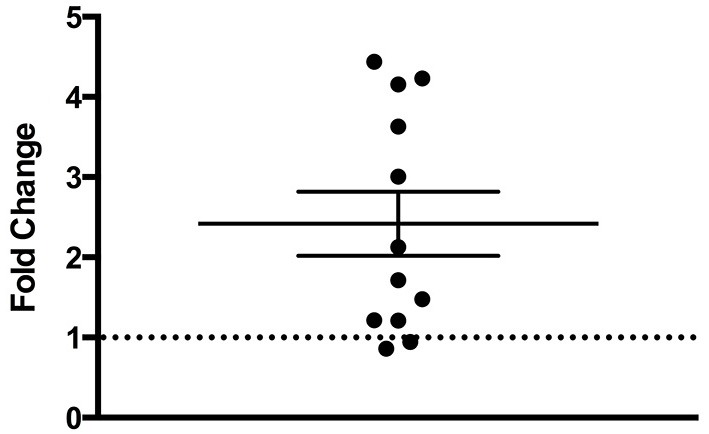
**Fold change in preferred room**. Subjects show a significant fold-increase in percent time spent in the preferred room during the final preference test compared to the initial preference test (*p* < 0.005). Individual data points are plotted with the Mean ± SEM. The dashed line at 1 indicates no fold change.

There was a statistically significant room preference (*t_11_* = 7.750, *p* < 0.0001) in comparing percent time spent in each room during the final preference test (Figure 3). To ascertain whether either room was more salient and capable of establishing a stronger preference, percent time spent on each side during the final preference test, independent of the specific US used (toys or books), was also compared. A paired *t*-test revealed both rooms produced equivalent scores, *t_11_* = 0.001580, *p* > 0.05 (Figure 4A). Additionally, to determine whether one US was capable of establishing a stronger preference, percent time spent in the room where the more engaging toys were presented was compared to percent time spent in the room containing the less engaging puzzles and books. No significant differences were found for US, *t_11_* = 0.3079, *p* > 0.05 (Figure 4B). These data demonstrate that both the US and CS are equally matched for saliency, and there is no preferential conditioning to one CS or US over the other. For the analysis of CPP scores, a one sample *t*-test revealed that the CPP scores were significantly different than the theoretical mean of 1 (if no conditioning occurred), *t_11_* = 3.538, *p* < 0.005 (Figure 5).

### Relationship Between CPP and MSEL

To determine whether developmental age and cognitive ability had a significant impact on performance in the CPP task, non-parametric spearman rank order correlations between each of the sub-scales (except gross motor because only three children had scores) and the early learning composite were performed. No correlations were found between any of the MSEL sub-scales or the early learning composite and conditioning scores (Table 2).

**Table 2:** **Spearman’s rank-order correlations for MSEL sub-scales and chronological age with CPP scores**.

	Spearman *r*	95% CI range	*p* value
MSEL Visual Reception with CPP	−0.4621	−0.8251–0.1710	*p* > 0.05
MSEL Fine Motor with CPP	−0.4727	−0.8294–0.1578	*p* > 0.05
MSEL Receptive Language with CPP	−0.5709	−0.8672–0.02373	*p* > 0.05
MSEL Expressive Language with CPP	−0.4939	−0.8378–0.1307	*p* > 0.05
MSEL Early Learning Composite with CPP	−0.2627	−0.7360–0.3831	*p* > 0.05
Chronological age with CPP	−0.3451	−0.7749–0.3030	*p* > 0.05

## Discussion

The main goal of this study was to successfully design a task that could provide reproducible measures of CPP in children without the need to engage language skills. A second goal was to develop a single visit paradigm that would be widely applicable in different research settings. To our knowledge, the present study provides the first data that demonstrates measurable and reproducible CPP in children. We focused on young subjects at preschool age, thus providing a strategy for early determination of internal affective state that can be paired with other forms of testing and questionnaires when neurodevelopmental disorders are diagnosed. We report here that subjects exhibit a significant place preference during the final preference test. Data also were collected to monitor any pre-conditioning biases and to determine the validity of the physical design of the castle, as well as experimental protocol for probing CPP. Our data suggested there was no strong preference for either room across the population of children studied, and therefore, no adaptations of the stimuli were necessary. These results strongly support this CPP design for use in young children. We believe that the specific conditioning stimuli used can be flexible, thereby being adaptive for potential cultural or ethnic factors.

The present study also is unique compared to previous human CPP studies by utilizing a CPP design that closely parallels that used in animals. Thus, an important element incorporated into the study was the initial preference test to address potential arena biases, which had not been done in the previous adult studies (Childs and de Wit, 2009; Molet et al., 2013; Astur et al., 2014). The absence of preference across the population in the initial preference test likely reflects that the demonstrated side preference during the final test is a direct result of pairing the CS with the US, and not an innate preference for the CS. The initial preference test also determines whether each CS is sufficiently salient for behavioral conditioning, and whether each CS is equivalently salient. Using the initial preference test scores, one can determine during the session the specific side of the arena in which the US should be presented. For example, if there is a strong preference for one room by a study subject, this would reduce the ability to demonstrate valid conditioning if the US is presented on the side that has an innate preference. In contrast, if the US is presented on the side that was not preferred during the initial preference test, then conditioning scores of course will tend to be inflated. With the population as a whole not showing a preference, and stimuli presentation remaining random and counter-balanced, as reported in the present study, then conditioning scores are likely to be more accurate or even conservative.

For this study, we had no prior knowledge the US that each child would find more rewarding. This likely contributes to the heterogeneity in preference scores, which reflect modest to robust conditioning. Thus, the scores presented in this initial study likely reflect a blunted CPP, and using a stimulus with known reinforcing/rewarding properties would allow us to show even greater conditioning scores.

The novel findings presented here not only reveal a unique behavioral task in children, but also provides a new technique for behavioral assessments in children with a broad range of cognitive capacities. The finding that none of the individual Mullen sub-scales correlated with CPP performance suggests that CPP might also be successful when used with populations with cognitive impairments. Moreover, this technique could also be useful for populations with language impairments or delays, as performance in this task does not depend upon expressive language. As such, the utility of this task is seen to reach far beyond the typical pediatric population, and can be a useful probe for understanding motivation and associative learning in children with neurodevelopmental disorders.

A consideration for future studies would be the addition of a behavioral response in these same children to confirm preference following conditioning. In the adult human CPP studies, participants verbally confirmed their preferred room following their behavioral task. While this study design was purposefully designed to be independent from expressive language, additional brief probes, such as a visual choice task following CPP, may be useful for verification of preference. Animal studies have revealed a complex interplay between the ventral striatum, dorsal hippocampus, and basolateral amygdala supporting CPP (Everitt et al., 1991; Ferbinteanu and McDonald, 2001). Human studies have revealed forebrain and striatal contributions for social reinforcement learning (Galvan et al., 2005; Jones et al., 2011). Future studies that probe physiological responses during CPP, and probe neurobiological circuit activation following successful CPP would provide mechanistic insight into whether similar neural circuitry underlies behavioral responses in the human CPP paradigm.

We recognize there is significant behavioral heterogeneity across typically developing children, even in other simple associative tasks, such as eyeblink conditioning (Reeb-Sutherland et al., 2011, 2012). Thus, the range of conditioning scores present in this task is most likely reflective of that heterogeneity. The CPP paradigm provides a continuous measure for describing associative learning and future social behavior phenotypes, and is thus more descriptive than a cut-off score, generating instead a dynamic range of possible behaviors. These descriptive data are critical for designing future studies that will focus on larger-scale application of CPP to clinical populations of children with neurodevelopmental disorders. Furthermore, these initial findings create future opportunities for using a variety of stimuli for conditioning in children. For example, sensory or social stimuli would be useful for revealing whether deficits in processing of specific stimuli in children are due to a lack of motivation for, or in contrast, a strong aversion to, those stimuli. Additionally, the CPP task allows for manipulations of saliency and complexity of the US, allowing for even greater understanding of reward and aversion thresholds. These paradigm adaptations will be useful in future studies of mechanisms that underlie differences in social and emotional behavior in typically or atypically developing children. Furthermore, while the current study has focused on very young children, the CPP task could be adapted for use in adolescents or even adults. The flexibility of this paradigm would allow for a comprehensive developmental study of reward and motivation, and the impact of maturation of cognitive control on performance in the CPP task (Casey et al., 2005; Somerville and Casey, 2010; Lourenco and Casey, 2013).

We note that there are numerous studies employing animal models to study human disorders, but the reverse—adaptation of well-regarded rodent models in studies to further understand human behavior—is the exception. Translational studies like this are necessary for understanding the biological underpinnings of human behavior and disorders. The use of well-conserved, behavioral probes that have clearly defined structure-function relationships in animals provides a unique opportunity to establish similar neurobiological relationships in children.

## Conflict of Interest Statement

The authors declare that the research was conducted in the absence of any commercial or financial relationships that could be construed as a potential conflict of interest.
